# Birthweight, childhood body size, and timing of puberty and risks of breast cancer by menopausal status and tumor receptor subtypes

**DOI:** 10.1186/s13058-022-01578-0

**Published:** 2022-11-11

**Authors:** Dorthe C. Pedersen, Britt W. Jensen, Anne Tjønneland, Zorana J. Andersen, Lene Mellemkjaer, Lise G. Bjerregaard, Julie Aarestrup, Jennifer L. Baker

**Affiliations:** 1 Center for Clinical Research and Prevention, Frederiksberg Hospital, Copenhagen University Hospital - Bispebjerg and Frederiksberg, Nordre Fasanvej 57, 2000 Frederiksberg, Denmark; 2 Danish Cancer Society Research Center, 2100 Copenhagen, Denmark; 3 Section of Environmental Health, Department of Public Health, University of Copenhagen, 1353 Copenhagen, Denmark

**Keywords:** Body mass index, Breast cancer, Childhood, Height, Menopausal status, Tumor receptor

## Abstract

**Background:**

Associations of birthweight, childhood body size and pubertal timing with breast cancer risks by menopausal status and tumor receptor subtypes are inconclusive. Thus, we investigated these associations in a population-based cohort of Danish women.

**Methods:**

We studied 162,419 women born between 1930 and 1996 from the Copenhagen School Health Records Register. The register includes information on birthweight, measured childhood weights and heights at the age of 7–13 years, and computed ages at the onset of the growth spurt (OGS) and at peak height velocity (PHV). The Danish Breast Cancer Cooperative Group database provided information on breast cancer (*n* = 7510), including estrogen receptor (ER), human epidermal growth factor receptor 2 (HER2) and menopausal status. Hormone replacement therapy use came from the Danish National Prescription Registry. Hazard ratios (HR) and 95% confidence intervals (CI) were estimated by Cox regression.

**Results:**

We found that birthweight was not associated with any breast cancer subtypes. While childhood BMI was not statistically significantly associated with ER+ tumors nor consistently with ER− tumors among pre-menopausal women, consistent inverse associations were found among postmenopausal women. At the age of 7 years, the HRs for postmenopausal ER+ and ER− tumors were 0.90 (95% CI 0.87–0.93) and 0.84 (95% CI 0.79–0.91) per BMI *z*-score, respectively. Similarly, childhood BMI was inversely associated with pre- and postmenopausal HER2− tumors, but not with HER2+ tumors. Childhood height was positively associated with both pre- and postmenopausal ER+ tumors, but not with ER− tumors. At the age of 7 years, the HRs for postmenopausal ER+ and ER− tumors were 1.09 (95% CI 1.06–1.12) and 1.02 (95% CI 0.96–1.09) per height *z*-score, respectively. In general, childhood height was positively associated with HER2+ and HER2− tumors among pre- and postmenopausal women. Ages at OGS and PHV were not associated with any breast cancer subtypes.

**Conclusions:**

We showed that a high BMI and short stature in childhood are associated with reduced risks of certain breast cancer subtypes. Thus, childhood body composition may play a role in the development of breast cancer.

**Supplementary Information:**

The online version contains supplementary material available at 10.1186/s13058-022-01578-0.

## Background

Female breast cancer is the most common cancer type in Denmark as well as globally and accounted for nearly 700,000 deaths in 2020 [1, 2]. Established risk factors for breast cancer include advancing age, early menarche, older age at first pregnancy, late menopause and use of hormone replacement therapy (HRT) [3–6]. Adult overweight (including obesity) is also linked with risks of breast cancer, although in a complex manner. Having overweight after the age of 18 years is consistently associated with reduced risks of pre-menopausal breast cancer [7–9], but with higher risks of postmenopausal breast cancer [3, 10]. Nevertheless, when breast cancer is further differentiated by tumor receptor subtype, such as estrogen receptor (ER) status and human epidermal growth factor receptor 2 (HER2) status, findings are sparse and less consistent [3]. For instance, a recent meta-analysis showed that adult body mass index (BMI) was not associated with either ER+ or ER− tumors in pre-menopausal women, whereas a positive association with ER+ tumors, but not with ER− tumors, was observed in postmenopausal women [11]. These mixed findings likely reflect the heterogeneity in the etiology of breast cancer subtypes.

It is established that birthweight, childhood BMI or adiposity (assessed by somatotype) and height, as well as the timing of puberty, are associated with risks of breast cancer [12–23]. Nevertheless, results from studies examining potential associations specifically by menopausal status and tumor receptor subtypes are inconsistent [17, 20–24]. As such, much remains unknown in relation to whether early life body size and pubertal timing relate to risks of breast cancer by menopausal status and tumor receptor subtypes. Therefore, we examined whether birthweight, childhood BMI and height as well as the timing of puberty were associated with risks of pre- and postmenopausal breast cancer overall and by ER and HER2 status in a large population-based cohort of Danish women.

## Methods

### Data sources

We used data from the Copenhagen School Health Records Register. This register includes computerized information on 200,978 girls (406,350 children) born from 1930 to 1996, which is virtually all children who ever attended a private or public school in Copenhagen, Denmark [25]. Because of a legally mandated school-based health program, the children underwent health examinations from the age of 7 to 13 years, where they had their height and weight measured by trained school doctors or nurses following standardized procedures. Until the 1970s, health examinations were conducted annually; thereafter, the number of examinations was reduced, and from 1983 onwards, children were typically only seen at school entry and exit. Birthweight was also obtained for children born in 1936 onwards, which was either recalled by the parents or transferred from the child’s health book at the first school health examination [25].

From the measured heights and weights, childhood BMI (kg/m^2^) was calculated. BMI and height were transformed into *z-*scores using internal references. Age at the onset of the growth spurt (OGS) and age at peak height velocity (PHV) were used as markers of pubertal timing and were derived from height measurements using validated methods, which required at least five measurements [26]. Therefore, pubertal timing measures were derived for women born between 1930 and 1969, when the health examinations were regularly performed throughout the school years.

Using a unique identification number, which was issued to all Danish citizens alive and living in Denmark on April 2, 1968, onwards, we linked the girls to the Danish Civil Registration System to obtain information on vital status [27]. Breast cancer diagnoses were identified through linkage with the Danish Breast Cancer Cooperative Group (DBCG) database. This database includes registrations of women with a first primary invasive breast cancer since 1977 together with detailed information on tumor characteristics, treatment and surgery as well as menopausal status [28]. The database was considered complete (> 95%) from the mid-1990s. Information on ER status by immunohistochemistry (IHC) staining has been available in the database since 1994. We defined tumors as ER+ if the IHC staining was ≥ 10% and as ER− if < 10% [29]. Information on HER2 status has been available from 2007 onwards and was assessed using IHC stains, which were scored as 0, 1+, 2  or 3+. Tumors that scored 0 and 1+ were defined as HER2-, and tumors that scored 3 + were defined as HER2+. Tumors that scored 2+ were further assessed by fluorescence in situ hybridization (FISH), and if the FISH ratio was ≥ 2.0, the tumor was defined as HER2+, otherwise it was defined as HER2− [30].

In addition to the DBCG database, we further identified women diagnosed with breast cancer prior to 1995 in the Danish Cancer Registry (with data available for this study from 1968 onwards) [31] to ensure that the study population only included women at risk of a first primary breast cancer. We obtained information on the use of HRT via linkage with the Danish National Prescription Registry [32]. This register contains all prescriptions dispended by Danish pharmacies since 1995, and we defined HRT using the following Anatomical Therapeutic Chemical (ATC) classification codes: G02BA03 (progestogen), G03C (estrogens), G03D (progestogens), G03F (estrogens and progestogens combined), G03HB01 (cyproterone and estrogens) and G03XC01 (raloxifen). Women were categorized as HRT users if they had redeemed at least two prescriptions of HRT products at the age of 45 years or older.

### Study population

In the analyses examining associations with pre- and postmenopausal breast cancer as well as ER status, we initiated follow-up on January 1, 1995. Of the 178,844 women with a valid personal identification number, we included women if they were alive and living in Denmark on the start date of follow-up or later and aged 18 years or older. We excluded women who were diagnosed with breast cancer prior to January 1, 1995 (*n* = 1825). Further, women with missing or outlying values of BMI or height at all childhood ages (*n* = 3551) were excluded (Additional file 1: Fig. S1). The analyses examining associations with birthweight were restricted to women born between 1936 and 1996 (*n* = 147,904; of whom 87% had information on birthweight), with a further exclusion of women with a birthweight outside the range of 2000–5500 g (*n* = 2591). The analyses examining associations with the ages at OGS and PHV were restricted to women born between 1930 and 1969 (*n* = 117,752; of whom 60% had information on ages at OGS and PHV).

The analyses examining associations with HER2 status began on January 1, 2007. After exclusions for death (*n* = 16,356), emigration (*n* = 5867), loss to follow-up (*n* = 9) or breast cancer prior to this date (*n* = 4196) and missing or outlying values of BMI and height (*n* = 3295), 149,121 women were available for these analyses.

Follow-up ended on the date of the first diagnosis of breast cancer, emigration, death or loss to follow-up on December 31, 2018, whichever came first. In the analyses by ER status, women with missing information on ER status (*n* = 338) or menopausal status (*n* = 16) were censored at breast cancer diagnosis. In the analyses by HER2 status, women with missing information on HER2 status (*n* = 117) or menopausal status (*n* = 14) were censored at breast cancer diagnosis.

### Statistical methods

Descriptive characteristics are presented as the median and the 5th and 95th percentiles or as percentages for categorical variables. Using Cox proportional hazards regression, we estimated the hazard ratios (HR) and 95% confidence intervals (CI) for the associations between birthweight (per 500 g, which corresponds to approximately 1 standard deviation in birthweight), childhood BMI and height *z* scores as well as ages at OGS and PHV, respectively, and risks of pre- and postmenopausal as well as ER and HER2 subtypes of breast cancer. Women who developed another breast cancer subtype than the one under investigation were censored at the date of diagnosis. For instance, in the analyses examining postmenopausal breast cancer, women who were diagnosed with pre-menopausal breast cancer were censored at the date of diagnosis.

In all analyses, age was the underlying time scale. Further, the analyses were stratified by three birth cohorts (1930–1939, 1940–1949 and 1950–1996) to allow the baseline hazard to differ by birth cohort. All analyses of postmenopausal breast cancer were adjusted for the use of HRT categorized as ever or never users.

Potential deviations from linearity in the associations of birthweight, childhood BMI and height *z* scores as well as ages at OGS and PHV, respectively, with the outcomes were assessed by comparing a restricted cubic spline model with a linear model using likelihood ratio tests. Limited indications of deviations from linearity in the associations were found; therefore, all results are presented linearly. We examined potential birth cohort effects by comparing models with and without interaction terms between the exposure of interest and birth cohort using likelihood ratio tests. Similarly, the proportional hazard assumption was tested using the likelihood ratio test to compare models with and without interaction terms between the exposure and categories of age at diagnosis. There were no consistent indications of birth cohort effects or violations of the proportional hazard assumptions. All statistical analyses were performed using Stata (version 15.1; StataCorp, College Station, TX).

## Results

This study included 162,419 women (Additional file 1: Fig. S1), and during 3.2 million women-years of observation time (median observation time, 24 years) a first primary breast cancer was diagnosed in 1272 pre-menopausal women and in 6238 postmenopausal women. In both pre- and postmenopausal women, ER+ tumors were the most common subtype accounting for 991 (78%) and 5226 (84%) of the cases, respectively. As expected, the median values of BMI and height increased with childhood age (Table 1). The median age at the OGS was approximately 1 year earlier than the age at PHV (Table 1). Women who were included in the analyses of birthweight and markers of puberty, respectively, and all women who potentially would have been included in these analyses (if all women have had information on these exposures available) were similar across a range of characteristics (Additional file 1: Supplementary Tables S1, S2). Similarly, women with missing information on ER and HER2 status, respectively, were similar to women with this information available (Additional file 1: Tables S3, S4).

**Table 1:** Characteristics of early life body size and pubertal timing for 162,419 girls from the Copenhagen School Health Records Register

Characteristic	*N*	Median	5th	95th
Birthweight (g)	125,352	3300	2500	4200
*BMI (kg/m*^*2*^*)*				
7 years	151,335	15.3	13.5	18.2
13 years	135,355	18.4	15.3	23.5
*Height (cm)*				
7 years	151,335	121.9	113.3	130.7
13 years	135,355	155.8	143.2	167.4
*Puberty markers (years)*				
Age at OGS	70,538	10.2	7.9	12.2
Age at PHV	70,538	11.8	9.6	13.6

*BMI* body mass index, *OGS* onset of the growth spurt, *PHV* peak height velocity

### Associations of early life body size and markers of puberty with breast cancer by menopausal status and ER status of tumors

**Fig. 1:**
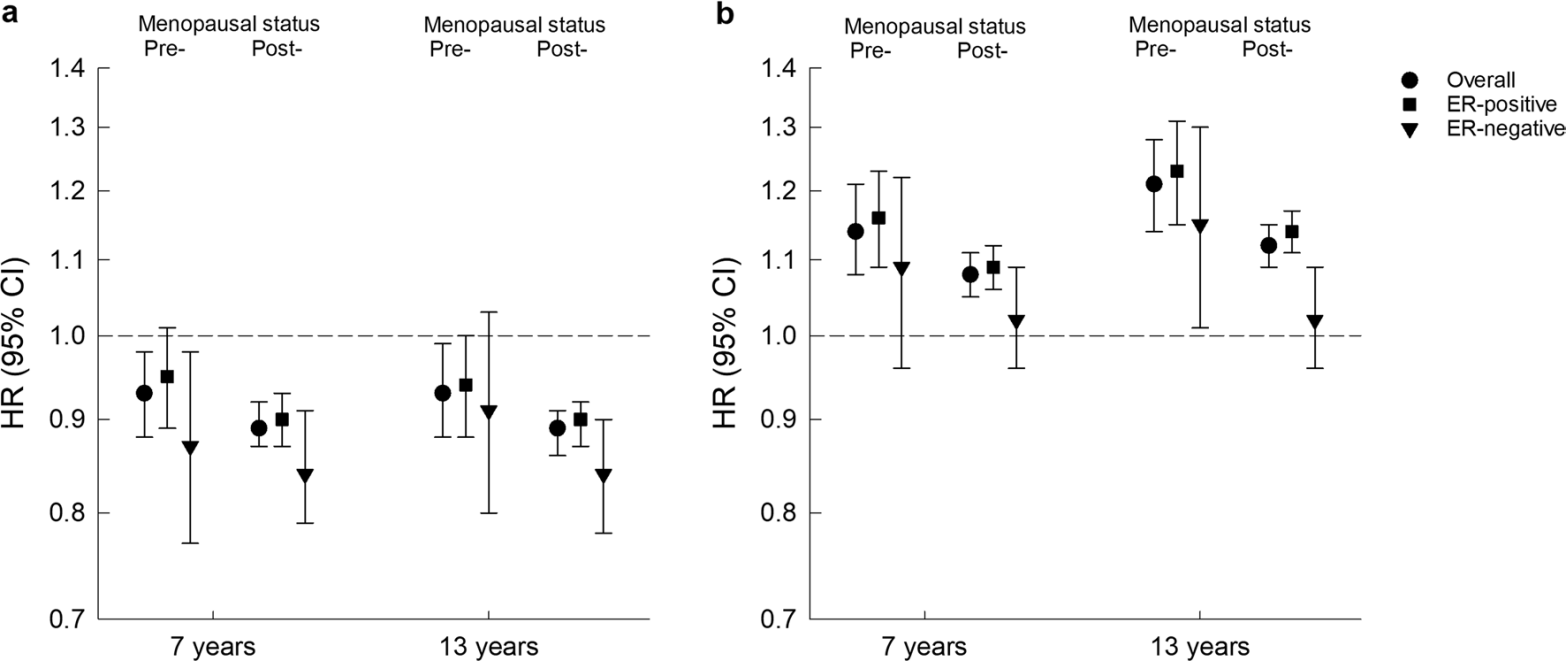
Associations between (**a**) childhood BMI (**b**) height and breast cancer by menopausal and ER status. Associations were estimated in girls from the Copenhagen School Health Records Register followed from January 1995. The Cox proportional hazards regression models were stratified by birth cohort. Postmenopausal analyses were adjusted for use of hormone replacement therapy. *BMI*, body mass index; *CI*, confidence interval; *ER*, estrogen receptor; *HR*, hazard ratio

No associations between birthweight and the risk of pre- and postmenopausal breast cancer overall or by ER status were found (Table 2). Childhood BMI at the age of 7 and 13 years was inversely associated with the risk of pre-menopausal breast cancer overall, but not significantly with ER+ tumors nor consistently with ER− tumors (Fig. 1 and Additional file 1: Table S5). In contrast, among postmenopausal women consistent inverse associations were observed between BMI at the age of 7 and 13 years and breast cancer overall as well as ER+ and ER− tumors (Fig. 1 and Additional file 1: Table S5). Childhood height at the age of 7 and 13 years was positively associated with the risk of pre-menopausal breast cancer overall and with ER+ tumors, but less consistent with ER− tumors (Fig. 1 and Additional file 1: Table S5). Among postmenopausal women, positive associations were observed between childhood height and risks of breast cancer overall and ER+ tumors, whereas no associations were observed with ER− tumors (Fig. 1 and Additional file 1: Table S5). Ages at OGS and PHV were not associated with either pre- or postmenopausal breast cancer overall or with the ER status of the tumors (Table 2).

**Table 2:** Associations between birthweight and puberty and breast cancer by menopausal and ER status

Characteristic	*N*	Pre-menopausal breast cancer						Postmenopausal breast cancer^a^					
		Overall		ER+		ER−		Overall		ER+		ER−	
		Cases	HR (95% CI)	Cases	HR (95% CI)	Cases	HR (95% CI)	Cases	HR (95% CI)	Cases	HR (95% CI)	Cases	HR (95% CI)
Birthweight, per 500 g	125,352	1120	1.03 (0.97–1.09)	865	1.03 (0.96–1.09)	255	1.03 (0.92–1.16)	4392	1.02 (0.99–1.05)	3699	1.03 (1.00–1.06)	693	0.97 (0.90–1.04)
*Puberty markers, per year*													
Age at OGS	70,538	710	0.95 (0.89–1.00)	566	0.94 (0.88–1.00)	144	0.98 (0.86–1.11)	3703	0.98 (0.96–1.01)	3104	0.98 (0.95–1.01)	599	1.01 (0.95–1.08)
Age at PHV	70,538	710	0.94 (0.89–1.00)	566	0.94 (0.88–1.01)	144	0.95 (0.83–1.08)	3703	0.99 (0.96–1.02)	3104	0.98 (0.96–1.01)	599	1.01 (0.94–1.08)

Associations were estimated in girls from the Copenhagen School Health Records Register followed from January 1995

*CI* confidence interval, *ER* estrogen receptor, *HR* hazard ratio, *OGS* onset of the growth spurt, *PHV* peak height velocity

^a^Adjusted for use of hormone replacement therapy

### Associations of early life body size and markers of puberty with breast cancer by menopausal status and HER2 status of tumors

Among the 149,121 women followed from January 2007, breast cancer was diagnosed in 533 pre- and 3720 postmenopausal women. The HER2− tumor was the most common subtype diagnosed in 450 (84%) pre-menopausal and in 3273 (88%) postmenopausal women.

Birthweight was not associated with breast cancer by HER2 tumor status in either pre- or postmenopausal women (Table 3). Similarly, no associations were observed between childhood BMI and HER2 + tumors in either pre- or postmenopausal women, whereas inverse associations were observed with both pre- and postmenopausal HER2− tumors (Table 3). In pre-menopausal women, childhood height was not associated with HER2 + tumors, but positively associated with HER2− tumors. However, among postmenopausal women, positive associations were found between childhood height and both HER2 + and HER2− tumors (Table 3). Ages at OGS and PHV were not associated with the HER2 status of tumors in either pre- or postmenopausal women (Table 3).

**Table 3:** Associations between birthweight, childhood body size, puberty and breast cancer by menopausal and HER2 status

Characteristic	N	Pre-menopausal breast cancer				Postmenopausal breast cancer^a^			
		HER2+		HER2−		HER2+		HER2−	
		Cases	HR (95% CI)	Cases	HR (95% CI)	Cases	HR (95% CI)	Cases	HR (95% CI)
Birthweight, per 500 g	117,493	76	0.94 (0.75–1.17)	392	1.00 (0.91–1.10)	346	0.95 (0.86–1.05)	2467	1.04 (1.00–1.08)
*BMI, per z-score*									
Age 7 years	139,117	79	0.91 (0.73–1.13)	431	0.88 (0.80–0.97)	416	0.93 (0.84–1.03)	3089	0.88 (0.85–0.92)
Age 13 years	123,166	68	0.87 (0.68–1.10)	386	0.87 (0.79–0.96)	432	0.92 (0.83–1.02)	3176	0.88 (0.84–0.91)
*Height, per z-score*									
Age 7 years	139,117	79	1.04 (0.83–1.29)	431	1.16 (1.05–1.28)	416	1.11 (1.01–1.23)	3089	1.08 (1.04–1.12)
Age 13 years	123,166	68	0.95 (0.74–1.20)	386	1.21 (1.10–1.34)	432	1.15 (1.05–1.27)	3176	1.13 (1.09–1.17)
*Puberty markers, per year*									
Age at OGS	63,565	35	1.17 (0.91–1.50)	219	0.96 (0.87–1.06)	255	0.95 (0.86–1.05)	2012	0.99 (0.96–1.02)
Age at PHV	63,565	35	1.23 (0.93–1.63)	219	0.98 (0.88–1.10)	255	0.92 (0.83–1.02)	2012	1.01 (0.97–1.05)

Associations were estimated in girls from the Copenhagen School Health Records Register followed from January 2007

*BMI* body mass index, *CI* confidence interval, *HR* hazard ratio, *HER2* human epidermal growth factor receptor 2, *OGS* onset of the growth spurt, *PHV* peak height velocity

^a^Adjusted for use of hormone replacement therapy

## Discussion

We found that a woman’s birthweight was not associated with pre- or postmenopausal breast cancer overall or by the ER or HER2 status of the tumors. Further, we showed that the higher the BMI in childhood, the lower the risks of pre- and postmenopausal breast cancer overall and of postmenopausal ER+ and ER− tumors as well as of pre- and postmenopausal HER2− tumors. Additionally, the taller a woman was in childhood, the higher her risks of pre- and postmenopausal breast cancer overall and of ER+ and HER2− tumors as well as postmenopausal HER2 + tumors. Further, we showed that ages at OGS and PHV were not associated pre- or postmenopausal breast cancer overall or when divided by tumor receptor subtype.

A large body of evidence supports an association between birthweight and breast cancer, especially at pre-menopausal ages. Meta-analyses showed that the risk estimates per 500 g of birthweight were 1.05 (95% CI 1.02–1.09) for pre-menopausal [3] and 1.06 (95% CI 1.02–1.09) for overall breast cancer [13]. However, many of the individual studies included in these meta-analyses were not statistically significant [3, 13], which corroborates our findings of no association between a woman’s birthweight and her later risk of pre- or postmenopausal breast cancer. While our null findings for breast cancer by ER status extend those from a smaller study based on the same data resource as in our study [12], few other studies have examined the association between birthweight and breast cancer by ER and HER2 status, but in general these also reported no associations [33, 34].

We found inverse associations between childhood BMI and pre- and postmenopausal breast cancer overall, postmenopausal ER+ and ER− tumors as well as pre- and postmenopausal HER2− tumors. While we did not identify other studies on childhood BMI, our overall findings on pre- and postmenopausal breast cancer extend those in another study based on the same data resource [14] and are in accord with studies on childhood adiposity (assessed by somatotype or weight relative to peers) [17–22, 24]. Previous studies on childhood adiposity and hormone receptor subtypes of breast cancer have yielded inconclusive findings [17, 20–22, 24]. In women (pre- and postmenopausal combined) with higher childhood adiposity, both lower [17] and no increased risks [21] of ER+ and ER− tumors have been reported when compared to women with lower childhood adiposity. Additionally, in studies examining these associations in pre- and postmenopausal women separately, no associations between childhood body fatness and pre-menopausal ER/progesterone (PR) status were reported [20], whereas a mix of inverse and no associations were reported with the ER and ER/PR status of tumors among postmenopausal women [20, 22, 24]. The inconsistent findings may be due to differences in how childhood adiposity was assessed and categorized, the number of women included in the studies as well as the assessment and definition of ER and PR status [20, 22, 24]. Investigations of childhood adiposity and HER2 status are limited, and we only identified two studies [17, 34]. Of the two, one reported inverse associations between childhood adiposity and risks of both HER2 + and HER2− tumors in pre- and postmenopausal women combined [17], whereas the other study reported an inverse association between adiposity and HER2− tumors, but not with HER2 + tumors, among postmenopausal women [34]. As such, our findings between childhood BMI and breast cancer by the tumor receptor subtypes among both pre- and postmenopausal women extend previous findings and substantially contribute to what is known in this research area.

The mechanisms underlying the inverse associations between childhood BMI and risks of breast cancer are speculative. Nevertheless, from the Continuous Update Project Expert Report 2018 from the World Cancer Research Fund/American Institute for Cancer Research it is evident that the timing and duration of excess weight are important for pre- and postmenopausal breast cancer risks [3]. In our study, we specifically looked at body size in late childhood, which is a period when the breast undergoes development [35]. Thus, it is plausible that excess adiposity at this time point could impact the composition of the developing breast and thereby later risks of breast cancer. In support of this, we have previously shown in a subset of this data resource that childhood BMI is inversely associated with mammographic density in postmenopausal women [36], which in turn is associated with lower risks of breast cancer overall [37].

We generally found positive associations between childhood height and breast cancer overall and by tumor receptor subtypes in both pre- and postmenopausal women, apart from HER2 + tumors in pre-menopausal women and ER− tumors in postmenopausal women. While our findings on pre- and postmenopausal breast cancer extend those from the previous study using this data resource [14], we did not identify other studies that examined the associations of childhood height with breast cancer by tumor receptor subtype. Nonetheless, our findings agree with those from a recent meta-analysis examining adult attained height in relation to tumors by ER status [38]. This study reported a positive association between attained height and ER+ tumors and no association with ER− tumors among pre- and postmenopausal women combined [38]. Further, our findings converge with those from another study, which reported that taller adult height was associated with increased risks of postmenopausal HER2 + and HER2− tumors [34].

Our findings of positive associations between childhood height and ER+ tumors are biologically plausible and may be related to growth-regulating hormones such as insulin-like growth factor-1 (IGF-1). Because of its role in promoting cell division and inhibiting apoptosis, it has been associated with breast cancer and ER+ tumors [39, 40]. In support of a causal role of IGF-1 in the etiology of ER+ tumors, a Mendelian randomization study among women in the UK Biobank recently showed that the genetically predicted IGF-1 level was associated with ER+ but not ER− tumors [41]. Thus, this emphasizes that height may serve as an important indicator of risk for ER+ tumors.

In this study, we found that ages at OGS and PHV were not associated with breast cancer when divided by menopausal status or by the tumor receptor subtype. Thus, our findings extend those from the previous study using the same data resource, although age at peak growth was derived using a different methodology than in this study [14]. Studies using age when reaching maximum height as a marker of pubertal timing reported no associations with neither pre- and postmenopausal breast cancer [42–44] nor postmenopausal tumors by ER status [23]. However, our findings are to some extent surprising given that age at menarche, another marker of pubertal timing, is generally reported to have inverse associations with pre- and postmenopausal breast cancer as well as hormone receptor subtypes [4, 45]. Nonetheless, as the ages at OGS and PHV capture the first phase of puberty and were derived from repeated measurements of height, it is challenging to make direct comparisons with studies that use recalled age at menarche, which is an indicator of the last phase of puberty.

Our study has several strengths. We included girls from a large population-based cohort of schoolchildren, of whom the majority were prospectively followed for 24 years through national registers and a national database. Further, the measurements of childhood height and weight were performed by trained staff, which ensures a low risk of measurement error and precludes bias associated with the recall of body size. As the coverage of the DBCG is nearly complete (> 95%), the risk of undetected cases is low. Additionally, because of the mandatory nature of the school health examination program for both public and private schools, and as healthcare access in Denmark is free and universal, the risk of selection bias into the cohort is minimal.

Our study also has certain limitations. HER2+ tumors consisted of both luminal B and HER2-enriched cases, and similarly, HER2− tumors were a mix of luminal A, luminal B and triple-negative cases [46]. As the prognosis is different for these specific subtypes, especially HER2-enriched and triple-negative cases having a worse prognosis than the luminal cases, it remains a limitation of our study that we were not able to distinguish among these subtypes in our analyses. Although we were able to account for HRT usage, information on other relevant covariates such as age at first birth and parity as well as lifestyle factors such as alcohol consumption was not available. As such, we cannot rule out potential bias by these factors in our estimates. Further, we cannot rule out the possibility of chance findings due to the performance of multiple analyses. The girls in the CSHRR are primarily Caucasian, and thus, our findings are likely generalizable to other Caucasian populations outside Denmark, but their generalizability to other ethnic populations requires further investigation.

## Conclusions

We found that birthweight was not associated with pre- and postmenopausal breast cancer. We also showed that a higher BMI in childhood was associated with lower risks of pre- and postmenopausal breast cancer overall, postmenopausal ER+ and ER− tumors as well as pre- and postmenopausal HER2− tumors. In contrast, the taller a woman was in childhood, the higher her risks of breast cancer overall, ER+, and HER2− tumors among both pre- and postmenopausal women as well as postmenopausal HER2 + tumors. Ages at OGS and PHV were not associated with breast cancer. Our findings highlight that childhood body size may be indicative of breast cancer risks and that an understanding of the underlying mechanisms is warranted to develop preventive efforts.

## Supplementary Information

**Additional file1**. **Table S1**: Characteristics and prevalence of pre- and postmenopausal breast cancer among women included in the analyses of birthweight and women who could potentially have been included in these analyses. **Table S2**: Characteristics and prevalence of pre- and postmenopausal breast cancer among women included in the analyses of puberty markers and women who could potentially have been included in these analyses. **Table S3**: Characteristics of women with and without information on ER status among women diagnosed with breast cancer after January 1995. **Table S4**: Characteristics of women with and without information on HER2 status among women diagnosed with breast cancer after January 2007. **Table S5**: Hazard ratios of the associations between childhood BMI and height and risks of overall, ER-positive and ER-negative breast cancers by menopausal status in girls from the Copenhagen School Health Records Register followed from January 1995. **Figure S1**: Flowchart of included and excluded women from the Copenhagen School Health Records Register (CSHRR).

## Data Availability

The code book and analytic code will be made available upon request to the corresponding author. The dataset used in this study is not publicly available due to being pseudo-anonymized, but access can be granted pending approval from the steering committee for these records following standardized access procedures.

## Abbreviations

ACT: Anatomical Therapeutic Chemical
CI: Confidence interval
CSHRR: Copenhagen School Health Records Register
DBCG: Danish Breast Cancer Cooperative Group
ER: Estrogen receptor
FISH: Fluorescence in situ hybridization
HER2: Human epidermal growth factor receptor 2
HR: Hazard ratio
HRT: Hormone replacement therapy
IGF-1: Insulin-like growth factor-1
IHC: Immunohistochemistry
OGS: Onset of the growth spurt
PHV: Peak height velocity
PR: Progesterone
