# Supplementary material for: Birthweight, childhood body size, and timing of puberty and risks of breast cancer by menopausal status and tumor receptor subtypes
Source: Breast Cancer Res. 2022 Nov 11;24:77. doi: 10.1186/s13058-022-01578-0 (PMC9652814; doi:10.1186/s13058-022-01578-0)
Supplement: Supplementary file 1 — Additional file1. Table S1: Characteristics and prevalence of pre- and postmenopausal breast cancer among women included in the analyses of birthweight and women who could potentially have been included in these analyses. Table S2: Characteristics and prevalence of pre- and postmenopausal breast cancer among women included in the analyses of puberty markers and women who could potentially have been included in these analyses. Table S3: Characteristics of women with and without information on ER status among women diagnosed with breast cancer after January 1995. Table S4: Characteristics of women with and without information on HER2 status among women diagnosed with breast cancer after January 2007. Table S5: Hazard ratios of the associations between childhood BMI and height and risks of overall, ER-positive and ER-negative breast cancers by menopausal status in girls from the Copenhagen School Health Records Register followed from January 1995. Figure S1: Flowchart of included and excluded women from the Copenhagen School Health Records Register (CSHRR). [file 13058_2022_1578_MOESM1_ESM.docx]

**Additional file 1: Supplementary information**

**Birthweight, childhood body size, and timing of puberty and risks of breast cancer by menopausal status and tumor receptor subtypes**

Dorthe C. Pedersen, Britt W. Jensen, Anne Tjønneland, Zorana J. Andersen, Lene Mellemkjaer, Lise G. Bjerregaard, Julie Aarestrup, Jennifer L. Baker

**Table of content**

[**Supplementary Table 1.** Characteristics and prevalence of pre- and post-menopausal breast cancer among women included in the analyses of birthweight and women who could potentially have been included in these analyses 2](#_Toc111206701)

[**Supplementary Table 2.** Characteristics and prevalence of pre- and post-menopausal breast cancer among women included in the analyses of puberty markers and women who could potentially have been included in these analyses 2](#_Toc111206702)

[**Supplementary Table S3.** Characteristics of women with and without information on ER status among women diagnosed with breast cancer after January 1995 3](#_Toc111206703)

[**Supplementary Table S4.** Characteristics of women with and without information on HER2 status among women diagnosed with breast cancer after January 2007 3](#_Toc111206704)

[**Supplementary Table S5.** Hazard ratios of the associations between childhood BMI and height and risks of overall, ER-positive and ER-negative breast cancers by menopausal status in girls from the Copenhagen School Health Records Register followed from January 1995 4](#_Toc111206705)

[**Supplementary Figure S1.** Flowchart of included and excluded women from the Copenhagen School Health Records Register (CSHRR) 5](#_Toc111206706)

## **Supplementary Table 1.** Characteristics and prevalence of pre- and post-menopausal breast cancer among women included in the analyses of birthweight and women who could potentially have been included in these analyses (birth years 1936-1996; *n* = 147,904)

|  | Included women |  | Potential population^a^ |
| --- | --- | --- | --- |
| Characteristic | *n* = 125,352 |  | *n* = 147,904 |
| BMI (kg/m^2^) at age 7 years, mean ± SD^b^ | 15.5 ± 1.5 |  | 15.5 ± 1.5 |
| BMI (kg/m^2^) at age 13 years, mean ± SD^c^ | 18.8 ± 2.6 |  | 18.8 ± 2.6 |
| Height (cm) at age 7 years, mean ± SD^b^ | 122.3 ± 5.2 |  | 122.2 ± 5.2 |
| Height (cm) at age 13 years, mean ± SD^c^ | 156.3 ± 7.2 |  | 156.1 ± 7.2 |
| Pre-menopausal breast cancer, *n* (%) | 1,120 (0.9) |  | 1,271 (0.9) |
| Age at diagnosis of pre-menopausal breast cancer (y), mean ± SD | 47.2 ± 5.8 |  | 47.0 ± 5.8 |
| Post-menopausal breast cancer, *n* (%) | 4,392 (3.5) |  | 5,219 (3.5) |
| Age at diagnosis of post-menopausal breast cancer (y), mean ± SD | 63.3 ± 6.9 |  | 63.7 ± 7.0 |

*BMI*, body mass index; *SD*, standard deviation

^a^ All women who potentially could have had information on birthweight

^b^ Women with BMI and height information at age 7 years: Included women *n* = 119,672; Potential population *n* = 138,331

^c^ Women with BMI and height information at age 13 years: Included women *n* = 104,028; Potential population *n* = 122,400

## **Supplementary Table 2.** Characteristics and prevalence of pre- and post-menopausal breast cancer among women included in the analyses of puberty markers and women who could potentially have been included in these analyses (birth years 1930-1969; *n* = 117,752)

|  | Included women |  | Potential population^a^ |
| --- | --- | --- | --- |
| Characteristic | *n* = 70,538 |  | *n* = 117,752 |
| BMI at age 7 years, mean ± SD^b^ | 15.3 ± 1.3 |  | 15.4 ± 1.4 |
| BMI at age 13 years, mean ± SD^c^ | 18.6 ± 2.3 |  | 18.6 ± 2.4 |
| Height at age 7 years, mean ± SD^b^ | 121.5 ± 5.1 |  | 121.4 ± 5.2 |
| Height at age 13 years, mean ± SD^c^ | 155.5 ± 6.9 |  | 155.0 ± 7.2 |
| Pre-menopausal breast cancer, *n* (%) | 710 (1.0) |  | 1,117 (1.0) |
| Age at diagnosis of pre-menopausal breast cancer (y), mean ± SD | 48.2 ± 4.8 |  | 48.2 ± 5.1 |
| Post-menopausal breast cancer, *n* (%) | 3,703 (5.3) |  | 6,237 (5.3) |
| Age at diagnosis of post-menopausal breast cancer (y), mean ± SD | 64.9 ± 7.6 |  | 65.2 ± 7.8 |

*BMI*, body mass index; *SD*, standard deviation

^a^ All women who potentially could have had information on markers of puberty

^b^ Women with BMI and height information at age 7 years information: Included women *n* = 68,386; Potential population *n* = 111,161

^c^ Women with BMI and height information at age 13 years information: Included women *n* = 70,331; Potential population *n* = 113,121

## **Supplementary Table S3.** Characteristics of women with and without information on ER status among women diagnosed with breast cancer after January 1995

|  | Information on ER status available | | |
| --- | --- | --- | --- |
|  | Yes |  | No |
| Characteristic | *n* = 7,510 (96%) |  | *n* = 338 (4%) |
| BMI at age 7 years, mean ± SD | 15.2 ± 1.3 |  | 15.1 ± 1.2 |
| BMI at age 13 years, mean ± SD | 18.3 ± 2.2 |  | 18.2 ± 2.1 |
| Height at age 7 years, mean ± SD | 121.6 ± 5.2 |  | 121.0 ± 5.1 |
| Height at age 13 years, mean ± SD | 155.5 ± 7.1 |  | 154.0 ± 7.0 |
| Age at diagnosis of breast cancer (y), mean ± SD | 62.1 ± 10.1 |  | 63.0 ± 10.5 |

*BMI*, body mass index; *SD*, standard deviation; *ER*, estrogen receptor

## **Supplementary Table S4.** Characteristics of women with and without information on HER2 status among women diagnosed with breast cancer after January 2007

|  | Information on HER2 status available | | |
| --- | --- | --- | --- |
|  | Yes |  | No |
| Characteristic | *n* = 4,253 (97%) |  | *n* = 117 (3%) |
| BMI at age 7 years, mean ± SD | 15.2 ± 1.2 |  | 15.1 ± 1.2 |
| BMI at age 13 years, mean ± SD | 18.3 ± 2.1 |  | 18.1 ± 2.1 |
| Height at age 7 years, mean ± SD | 121.8 ± 5.2 |  | 120.8 ± 5.0 |
| Height at age 13 years, mean ± SD | 155.8 ± 7.2 |  | 154.4 ± 6.8 |
| Age at diagnosis of breast cancer (y), mean ± SD | 65.0 ± 10.3 |  | 67.0 ± 9.0 |

*BMI*, body mass index; *SD*, standard deviation; *HER2*, human epidermal growth factor receptor 2

# **Supplementary Table S5.** Hazard ratios of the associations between childhood BMI and height and risks of overall, ER-positive and ER-negative breast cancers by menopausal status in girls from the Copenhagen School Health Records Register followed from January 1995

|  |  |  |  | Pre-menopausal breast cancer | | | | | | | |  | Post-menopausal breast cancer^a^ | | | | | | | |
| --- | --- | --- | --- | --- | --- | --- | --- | --- | --- | --- | --- | --- | --- | --- | --- | --- | --- | --- | --- | --- |
|  |  |  |  | Overall | |  | ER+ | |  | ER- | |  | Overall | |  | ER+ | |  | ER- | |
| Characteristic |  | N |  | Cases | HR (95% CI) |  | Cases | HR (95% CI) |  | Cases | HR (95% CI) |  | Cases | HR (95% CI) |  | Cases | HR (95% CI) |  | Cases | HR (95% CI) |
| BMI, *z* score | |  |  |  |  |  |  |  |  |  |  |  |  |  |  |  |  |  |  |  |
| Age 7 years | | 151,335 |  | 1,228 | 0.93 (0.88-0.98) |  | 959 | 0.95 (0.89-1.01) |  | 269 | 0.87 (0.77-0.98) |  | 5,847 | 0.89 (0.87-0.92) |  | 4,899 | 0.90 (0.87-0.93) |  | 948 | 0.84 (0.79-0.91) |
| Age 13 years | | 135,355 |  | 1,172 | 0.93 (0.88-0.99) |  | 921 | 0.94 (0.88-1.00) |  | 251 | 0.91 (0.80-1.03) |  | 5,980 | 0.89 (0.86-0.91) |  | 5,013 | 0.90 (0.87-0.92) |  | 967 | 0.84 (0.78-0.90) |
| Height, *z* score | |  |  |  |  |  |  |  |  |  |  |  |  |  |  |  |  |  |  |  |
| Age 7 years | | 151,335 |  | 1,228 | 1.14 (1.08-1.21) |  | 959 | 1.16 (1.09-1.23) |  | 269 | 1.09 (0.96-1.22) |  | 5,847 | 1.08 (1.05-1.11) |  | 4,899 | 1.09 (1.06-1.12) |  | 948 | 1.02 (0.96-1.09) |
| Age 13 years | | 135,355 |  | 1,172 | 1.21 (1.14-1.28) |  | 921 | 1.23 (1.15-1.31) |  | 251 | 1.15 (1.01-1.30) |  | 5,980 | 1.12 (1.09-1.15) |  | 5,013 | 1.14 (1.11-1.17) |  | 967 | 1.02 (0.96-1.09) |

*BMI*, body mass index; *CI*, confidence interval; *ER*, estrogen receptor; *HR*, hazard ratio

^a^ Adjusted for use of hormone replacement therapy


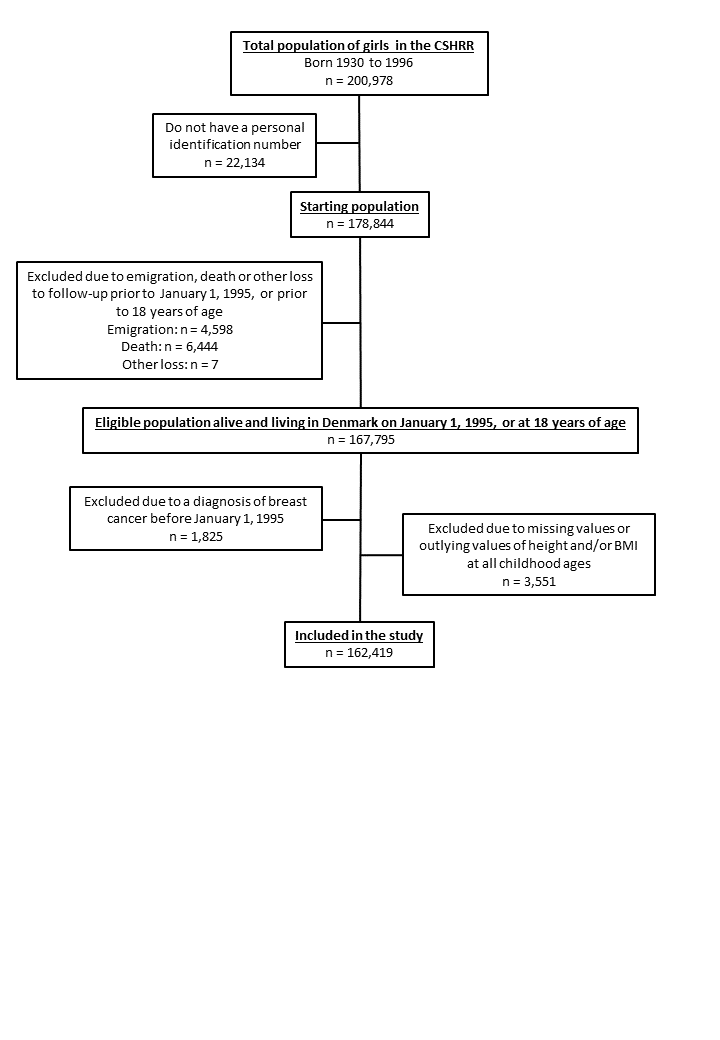


# **Supplementary Figure S1.** Flowchart of included and excluded women from the Copenhagen School Health Records Register (CSHRR)
